# Transient chaos - a resolution of breakdown of quantum-classical correspondence in optomechanics

**DOI:** 10.1038/srep35381

**Published:** 2016-10-17

**Authors:** Guanglei Wang, Ying-Cheng Lai, Celso Grebogi

**Affiliations:** 1 School of Electrical, Computer, and Energy Engineering, Arizona State University, Tempe, Arizona 85287, USA; 2 Department of Physics, Arizona State University, Tempe, Arizona 85287, USA; 3 Institute for Complex Systems and Mathematical Biology, King’s College, University of Aberdeen, Aberdeen AB24 3UE, UK

## Abstract

Recently, the phenomenon of *quantum-classical correspondence breakdown* was uncovered in optomechanics, where in the classical regime the system exhibits chaos but in the corresponding quantum regime the motion is regular - there appears to be no signature of classical chaos whatsoever in the corresponding quantum system, generating a paradox. We find that transient chaos, besides being a physically meaningful phenomenon by itself, provides a resolution. Using the method of quantum state diffusion to simulate the system dynamics subject to continuous homodyne detection, we uncover transient chaos associated with quantum trajectories. The transient behavior is consistent with chaos in the classical limit, while the long term evolution of the quantum system is regular. Transient chaos thus serves as a bridge for the quantum-classical transition (QCT). Strikingly, as the system transitions from the quantum to the classical regime, the average chaotic transient lifetime increases dramatically (faster than the Ehrenfest time characterizing the QCT for isolated quantum systems). We develop a physical theory to explain the scaling law.

The quantum-classical correspondence is a fundamental and fascinating problem in physics. For a specific physical process in a quantum system, if a large number of energy levels are involved (e.g., in the high energy regime), the evolution of the expected values of the observables will be governed by the classical Newtonian dynamics. This is the usual quantum-classical correspondence. Exceptions can occur when only a few lower energy levels are involved, e.g., at low temperatures, such that the quantum features of the ground state are manifested on a macroscopic scale^1^, leading to fascinating phenomena such as Bose-Einstein condensation, superconductivity, and superfluids. In this paper, we report our discovery of transient chaos as a natural paradigm to explain the recently discovered phenomenon of the breakdown of quantum-classical correspondence in optomechanics.

A prototypical optomechanical system consists of an optical cavity with a fixed mirror and a nanoscale, mechanically movable cantilever, as shown schematically in Fig. 1. The basic physics is that the radiation pressure from the optical field changes the position of the movable mirror, which in return modulates the resonance frequency of the optical cavity, leading to a coupling between the optical and mechanical degrees of freedom^2^,^3^. In addition to this prototypical setting, alternative configurations for realizing the optical-mechanical coupling exist, such as those based on the whispering-gallery modes^4^, microtoroid^5^ and microsphere^3^ resonators. Optomechanics is thus not only fundamentally important, as it provides a setting to understand the physics of optical-mechanical interactions^3^,^6^,^7^, but also practically significant with applications ranging from ultra-precision measurements^2^,^8^,^9^, light-matter entanglement^10^,^11^,^12^, mechanical memory^13^, tunable optical coupler^14^, classical state preparation through squeezing^15^,^16^, optical transparency^17^, and photon shuttling^18^ to creation of nonclassical light^19^,^20^ and cooling of microscopic or mesoscopic objects^21^,^22^. The classical equations of motion of an optomechanical system are nonlinear, rendering possible chaotic behaviors^23^,^24^.

In a recent work^25^, it was demonstrated that, in the classical regime the system exhibits chaos, but in the corresponding quantum regime the motion becomes regular and no signatures of chaos appear to exist. This is the so-called *quantum-classical correspondence breakdown* in optomechanics. A conventional approach to studying the correspondence is to compare the quantum Wigner function distribution with the classical phase space distribution^20^,^26^,^27^, both being average quantities. However, a recent work^28^ demonstrated an optimal state estimation for cavity optomechanical systems through Kalman filtering, which allows us to obtain the conditional system state in the presence of experimental noise. In addition, observation of quantum trajectories obeying quantum state diffusion through heterodyne detection in a coupled system between a superconducting qubit and an off-resonant cavity was reported^29^, as well as other types of quantum trajectories^30^,^31^,^32^,^33^,^34^,^35^,^36^,^37^,^38^,^39^,^40^. Thus, rather than focusing on the average properties of the system, we study the individual quantum trajectories of the system as related to the continuous weak measurement to probe into the quantum-classical correspondence breakdown.

Our aim is to uncover, through systematic classical and quantum simulations, the dynamical and physical mechanisms responsible for the breakdown phenomenon. The standard treatment^3^ of an optomechanical system consists of quantizing the cavity optical field and the oscillations of the cantilever as two mutually interacting quantum boson fields while treating the driving laser field classically. Dissipation associated with the optical and mechanical fields can be incorporated into the quantum Langevin equations from the quantum input-output theory^41^ or by solving the quantum Master equation with the Lindblad operators. When chaos occurs in the classical limit, the system is typically in a high energy state with hundreds of photons and phonons, rendering infeasible direct simulation of the quantum Master equation. An effective framework is the method of quantum state diffusion (QSD), which generates quantum trajectories to approximate the time evolution as governed by the quantum Master equation^42^,^43^,^44^. The QSD method has been instrumental to homodyne detection and the study of quantum-classical correspondence in dissipative quantum chaos^45^,^46^,^47^. Here, using the QSD method, we calculate the dynamical trajectories of the system in the quantum regime. Our computations extending to the long time scales (which were not attempted in previous works) suggest that transient chaos^48^ associated with quantum trajectories is ubiquitous. (To our knowledge, in spite of reports of chaos^3^,^23^,^24^, there were no prior results of transient chaos in optomechanical systems). In particular, before approaching a regular final state, the quantum system exhibits a behavior that is consistent with the classical chaotic behavior. Thus, in short and in long time scales, the time evolutions of the system in the quantum regime would appear to be chaotic and regular, respectively. This means that, in short time scales a quantum-classical correspondence does exist, but its breakdown occurs in the long time limit. A striking finding is that, as the classical regime is approached, the average transient lifetime increases dramatically (faster than the Ehrenfest time - see Discussion). As the quantum system becomes “more classical,” the quantum-classical correspondence holds significantly longer, providing a natural resolution for the breakdown phenomenon.

## Results

### Hamiltonian

In the rotating frame of the driving laser field, the Hamiltonian of a generic optomechanical system is^3^:


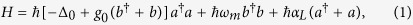


where *a*^†^ and *a* are the creation and annihilation operators for the optical field, *b*^†^ and *b* are the corresponding phonon operators for the mechanical cantilever, Δ_0_ = *ω*_*d*_ − *ω*_*cav*_ is the detuning between the driving laser and the optical cavity field, and *ω*_*m*_ is the resonant frequency of the mechanical mode. The quantity *α*_*L*_ is the classical amplitude of the driving laser field, which is related to its power *P* through |*α*_*L*_|^2^ = 2*κP*/(*ħω*_*d*_), where *κ* is the quality factor of the optical cavity. The basic physics behind the optomechanical coupling^49^ is that a change in the position of the cantilever, which is proportional to (*b*^†^ + *b*), can lead to a change in the resonant frequency of the optical field with a strength factor *g*_0_, where 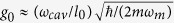, with *l*_0_ being the nominal cavity length.

### Calculation of classical trajectories

A conventional approach to investigating the dynamics of an optomechanical system is to use the quantum input-output theory^41^ to obtain the standard quantum Langevin equations in the Heisenberg picture. While dissipation and fluctuations of the photon and phonon fields have been taken into account, these are operator equations with stochastic fluctuations. In the classical limit (*ħ* → 0), i.e., bad cavity limit, the quantum correlations between the operators are negligible as compared with their averages, so we have^12^ 〈(*b*^†^ + *b*)*a*〉 ≈ 〈*b*^†^ + *b*〉〈*a*〉. Under this approximation, the operator equations can be replaced by those for the corresponding mean values, leading to the semiclassical Langevin equations. The deterministic dynamics of the system can be assessed by neglecting the small fluctuations in the photon and phonon fields. The resulting deterministic equations are:


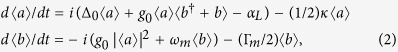


where Γ_*m*_ is the mechanical dissipation rate. A property of the classical equations is that, if *b* and *a* are replaced by *g*_0_*b* and *a*/*α*_*L*_, respectively, the resulting equations contain the parameter 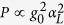, where *g*_0_ and *α*_*L*_ no longer appear as individual parameters. If other parameters are kept constant, the dynamics of the classical system is solely determined by the power of the driving laser field, i.e., *P*, with *g*_0_ and *α*_*L*_ as scaling factors. Intuitively, this can be understood by noting that, when a quantum system approaches its classical limit, *ħ* vanishes so that the quantum strength factors 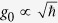 and 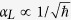 (both containing *ħ*) are reduced into a single parameter *P* that does not contain *ħ*. However, in the stochastic Langevin equations, the strengths of the quantum fluctuations associated with the photon and phonon fields are proportional to *g*_0_ and 1/*α*_*L*_, respectively. In the moderate and deep quantum regimes away from the classical limit, as *g*_0_ is increased, the deterministic Langevin equations are less meaningful due to the more pronounced quantum fluctuations.

**Figure 2:**
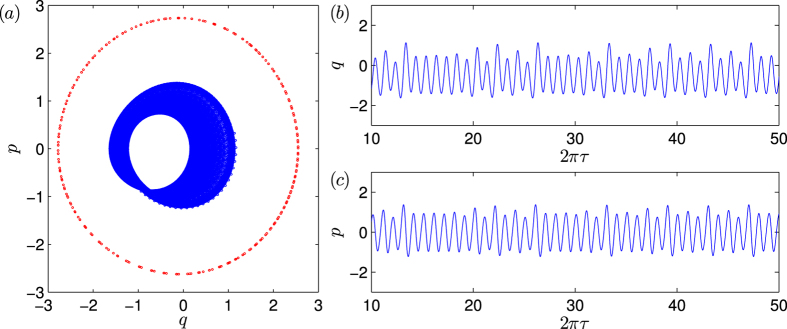
From the deterministic classical equation, (**a**) a representative chaotic trajectory and (**b,c**) the corresponding time series for *q* and *p*. The dashed circle in (**a**) indicates a coexisting periodic attractor.

The classical equations are nonlinear, so chaos can arise, as uncovered in previous experimental^23^,^24^ and theoretical^25^,^50^,^51^ works. To demonstrate the chaotic behavior, we use the same parameter setting as in the recent work of Bakemeier *et al*.^25^: *κ*/*ω*_*m*_ = 1.0, Γ_*m*_/*ω*_*m*_ = 10^−3^, Δ_0_/*ω*_*m*_ = −0.7, and 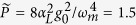. Figure 2(a) shows a representative chaotic orbit in the two-dimensional subspace of the variables 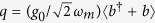 and 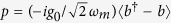, where the evolution time *τ* is made dimensionless through *τ* ≡ *ω*_*m*_*t*. The corresponding chaotic time series is shown in Fig. 2(b,c).

### Calculation of quantum trajectories

The quantum evolution of the optomechanical system can be calculated by using the quantum Master equation, which incorporates the effects of photon and phonon dissipation through the Lindblad operators. In particular, at zero temperature the quantum Master equation is^3^,^52^


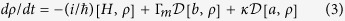


where the Lindblad operator is given by


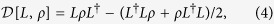


and *L* stands for either *a* or *b*. The quantum Master equation describes the time evolution of an ensemble of identical quantum systems. The dimension of the optomechanical system is (*N*_*a*_*N*_*b*_)^2^, where *N*_*a*_ and *N*_*b*_ denote the highest photon and phonon Fock states, respectively. An approach to reducing the dimension to (*N*_*a*_*N*_*b*_) is to “unravel” the deterministic quantum Master equation through the stochastic wavefunction equation for quantum trajectories^42^,^43^,^44^,^53^. The deterministic property is retained through the ensemble average of many realizations of the system starting from the same initial condition. Among the many unraveling schemes for generating quantum trajectories, the QSD approach is convenient and efficient with results that can be related to the record of homodyne detection, an important measurement tool in optomechanics^28^. The QSD equation is given by^54^,^55^


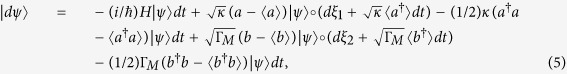


where 〈*O*〉 = 〈*ψ*|*O*|*ψ*〉 is the expectation value of operator *O* for the specific wave function |*ψ*〉. The QSD equation (5) is in fact a Stratonovich type of stochastic equations. (The Ito form of QSD has also been established and widely used^42^,^43^,^44^,^45^,^56^). In the QSD equation, the terms *dξ*_*j*_ (*j* = 1, 2) are complex Gaussian white noise for the photon and phonon fluctuations, which satisfy


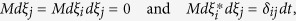


where *M* stands for the ensemble average. The density operator can be reconstructed through the mean over the projectors of the ensemble quantum states


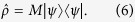


**Figure 3:**
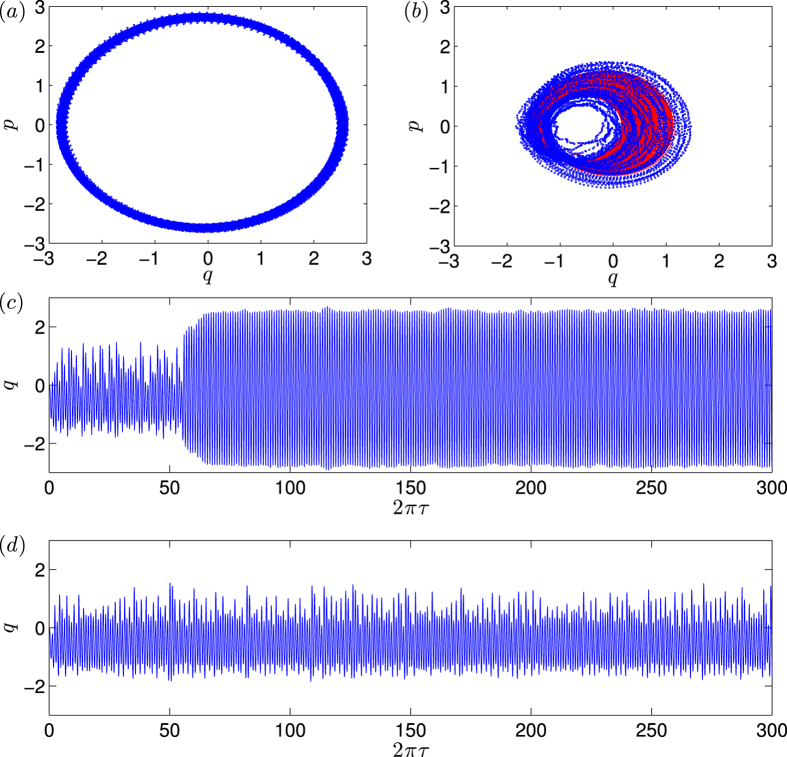
For *g*_0_/*ω*_*m*_ = 0.1, (**a**) an asymptotic quantum trajectory calculated from the QSD method, (**b**) the quantum trajectory in the transient phase, overlapped with the corresponding classical trajectory, (**c**) the corresponding time series. The asymptotic quantum trajectory is regular, in spite of the quantum fluctuations. However, the transient quantum trajectory is chaotic and coincides well with the classical trajectory (gray). (**d**) An example of a very long chaotic transient in the quantum regime for *g*_0_/*ω*_*m*_ = 0.05.

In an optomechanical system, the quantum effects can be characterized by the parameters 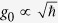 and 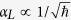. Figure 3(a) shows, for *g*_0_/*ω*_*m*_ = 0.1, a typical quantum trajectory calculated from the QSD equation in the (*q*, *p*) plane. This is a periodic, limit-cycle trajectory, despite being noisy due to the quantum fluctuations. The corresponding time series *q*(2*πτ*) is shown in Fig. 3(c). Since the value of the laser power *P* is fixed, the corresponding classical behavior is that shown in Fig. 2, which is chaotic. The remarkable phenomenon is that, the quantum trajectory in Fig. 3(a) is characteristically different from the classical trajectory in Fig. 2(a): the former is regular while the latter is chaotic! This is the recently discovered phenomenon of quantum-classical correspondence breakdown in optomechanical systems^25^.

### Transient chaos in the quantum regime

We find that the breakdown can be naturally viewed as a manifestation of transient chaos. We note from Fig. 3(c) that, before the periodic quantum state is reached, there is a relatively short time interval during which the quantum evolution is characteristically different, which is a transient phase. The quantum trajectory of the system in the transient phase is shown in Fig. 3(b), which appears chaotic. The striking finding is that, the transient quantum trajectory is remarkably consistent (in fact coincides) with the corresponding classical trajectory (the red background trajectory in Fig. 3(b), which overlaps with the quantum trajectory almost completely). As we tune the parameter *g*_0_/*ω*_*m*_ towards the classical regime, the duration of the transient phase increases. The extreme situation is that the transient time becomes so long that the system stays in a chaotic state for any practical time. An example is shown in Fig. 3(d) for *g*_0_/*ω*_*m*_ = 0.05.

**Figure 4:**
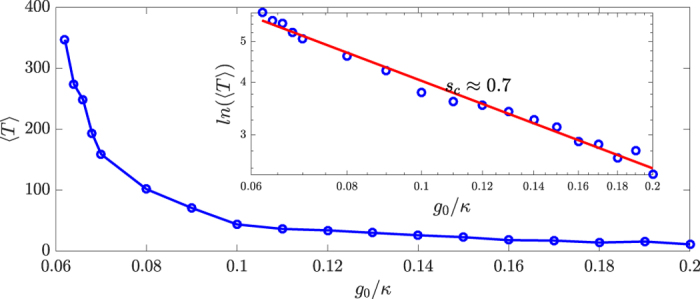
QSD Results. Dependence of average chaotic transient lifetime, 〈*T*〉, on *g*_0_ on a linear-linear plot and on a double logarithmic versus logarithmic scale (inset). All points are result of averaging 100 QSD realizations.

How does the average chaotic transient lifetime 〈*T*〉 depend on the quantum strength parameter *g*_0_? Here, the quantity 〈*T*〉 is the average time required for the system to transition from a chaotic attractor in the classical limit to a coexisting periodic attractor in the quantum regime, as induced by quantum fluctuations. For example, for a specific trajectory in Fig. 3(c), the transition occurs at 2*πτ* ≈ 55, so the transition time is *T* = 55/2*π*. (Note that the evolution time *τ* is made dimensionless through *τ* ≡ *ω*_*m*_*t*). From Fig. 3(c), we also see a dramatic change in the amplitude before and after the transition, and this can be exploited for efficiently computing the average transition time from a large number of quantum trajectories. In general, the time from the beginning to the end of the transition can be neglected as compared with the typically long transient time, especially when the effective Planck constant is reduced. As shown in Fig. 4, as *g*_0_ is decreased so that the quantum effect becomes progressively weaker, 〈*T*〉 increases dramatically. A qualitative explanation for Fig. 4 is the following. The classical trajectories are calculated from the deterministic, semiclassical Langevin equation in the Heisenberg picture with dissipation, where quantum fluctuations are neglected. The quantum trajectories are obtained from the QSD method, an unraveling of the general quantum Master equation in the Schrödinger picture using the Lindblad operators. The quantum fluctuations in QSD not only play the role of noise in the classical deterministic system, but more importantly, they can induce characteristic changes in the system dynamics. Say we fix the laser power so that the classical dynamics remains chaotic. What will happen when the quantum effects (fluctuations) become increasingly pronounced? Mathematically, as *g*_0_ is increased, it is necessary to decrease *α*_*L*_ to keep the driving laser power constant. This effectively enhances the ratios 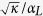 and 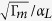 in the QSD equation, which are the relative noise-to-driving ratios. As noise becomes more pronounced, the probability that the system can stay in the deterministic chaotic set is decreased, reducing the chaotic transient lifetime.

To further test the proposition that noise or quantum fluctuations can drive the quantum system away from the classical chaotic invariant set, we calculate the quantum trajectories but with the noise term excluded. We find that, without random fluctuations, the quantum trajectories follow the classical chaotic set *all the time*. This result confirms that it is the quantum fluctuations which eventually drive the quantum trajectories out of the classical chaotic set, generating transient chaos. The weaker the quantum fluctuations, the longer the average transient lifetime will be. The quantum-classical transition is thus induced by quantum fluctuations, which resembles the phenomenon of noise-induced transition in classical systems that can be treated using the classical Kramer rate theory^57^. The transient chaos associated with quantum-classical transition is also relevant to the quantum activation process^58^, a transition process induced by noise between coexisting asymptotic states in a quantum system. We remark that, in a related work^59^, it was reported that quantum isoperiodic stable structures can be retained by the information from the classical isoperiodic stable structures in presence of noise.

### Scaling of transient lifetime and physical understanding

The Kramer theory or the quantum activation theory stipulates that the escape rate *κ_s_* generally follows the scaling as


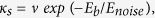


where *E*_*b*_ denotes the threshold energy for activation, *ν* is a prefactor, and *E*_*noise*_ is the strength of the fluctuation, e.g., on the order of 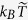 due to the thermal environment or *ħω* in the deep quantum regime, where 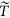 represents temperature. At low temperatures, the quantum fluctuations are dominated by the zero-point energy.

Figure 4 shows the relation between the average chaotic transient lifetime 〈*T*〉 and the magnitude *g*_0_ of the quantum fluctuations on a double logarithmic scale. The relation can be well fitted by a straight line, as shown in the inset of Fig. 4, which indicates the scaling law:


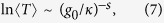


where −*s (s* > 0) is the slope of the linear fit. The scaling law is characteristic of superpersistent chaotic transients in nonlinear dynamical systems^60^,^61^,^62^,^63^,^64^. The physical meaning is that, as the quantum fluctuations are reduced so that the classical description becomes more accurate, the chaotic behavior becomes significantly more persistent in that its lifetime increases faster than the Ehrenfest time.

To better understand the scaling behavior of the average transient lifetime, we exploit the quantum Langevin equations:


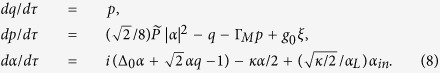


**Figure 5:**
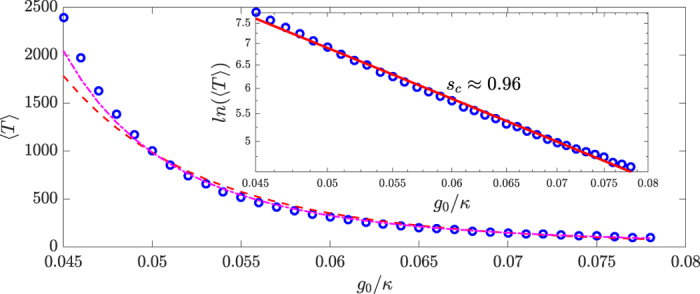
Results from classical Langevin equation. (**a**) Dependence of the average chaotic transient lifetime, 〈*T*〉, on *g*_0_ on a linear-linear plot. Inset: the same plot but on a double logarithmic versus logarithmic scale. The magenta dash-dot curve is a fit of the superpersistent chaotic transients behavior while the red dash curve is a fit of the Ehrenfest scaling. In the inset the red straight curve shows the slope of the supperpersistent chaotic transients behavior is about −*s*_*c*_ ≈ −0.96. All points are result of averaging 10000 Langevin equation realizations.

In general, a Langevin equation can be analyzed using the corresponding Fokker-Planck equation, where the stochastic component of the former contributes to the diffusion term in the evolution of the probability distribution of the latter. For the Fokker-Planck equation, a general solution cannot be written down explicitly except for one-dimensional systems. In this case, the steady state distribution has the form 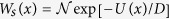, where *U*(*x*) is the effective potential, *D* is the noise amplitude proportional to 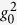, and 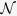 is a normalization constant. The mean first passage time over a barrier, i.e., the diffusion time from a local minimum *U*(*a*) over a saddle point *U*(*b*), obeys the following scaling law^65^ with *D*: *T*_*MFP*_ ∝ *e*^[*U*(*b*)−*U*(*a*)]/*D*^. However, to predict the exact form of the scaling law from the general multivariable Fokker-Plack equation is difficult. An alternative is to calculate the average chaotic transition lifetime (or the mean first passage time) from the Langevin equations. The results are shown in Fig. 5. Due to the relative simplicity of the Langevin equation as compared with the QSD equation, it is possible to probe more deeply into the classical regime with much longer transition lifetime. We find that, in the *g*_0_ regime where both types of results are available, the agreement is excellent. In particular, solutions of the Langevin equation gives


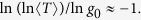


In Fig. 5(a), we show the fitting curve of the Ehrenfest scaling (red dash) as well as the superpersistent chaotic transition behavior (magenta dash-dot). For the Ehrenfest scaling, we use the least-squares method to fit 〈*T*〉 = *C*_0_ ⋅ *g*^−*δ*^ on a double logarithmic scale. For the superpersistent scaling, it is not straightforward to fit the relation 
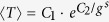
. We thus set *C*_1_ = 1 and fit the simulation results in terms of ln[ln(〈*T*〉)] versus ln *g*_0_. We see that the magenta curve fits better than the red curve, especially in the middle region. For small values of *g*_0_/*ω*_0_, the Ehrenfest scaling exhibits larger deviations from the simulation results as compared with the superpersistent transient scaling.

For the QSD results (Fig. 4), we estimate the slope of the fitting line of ln ln(〈*T*〉) with ln *g*_0_ and obtain the absolute value of about 0.7, which is smaller than the result from the Langevin equation. There can be multiple reasons for the difference. For example, for a large value of *g*_0_/*ω*_0_, the trajectories tend to approach the periodic attractor from the beginning. However, the transition process takes time, so the state at an arbitrary instant of time during the transition is actually recorded. When the transition time is comparable with the transient time, error can occur. Considering that our system is higher than one dimensional and the simulations were done with the full quantum state diffusion equation, the difference in the slope may not be unreasonable. In particular, in high dimensions the slope should have a smaller absolute value because of the existence of more “paths” to cross the saddle point (there is only one route in one dimension), facilitating the transition.

**Figure 6:**
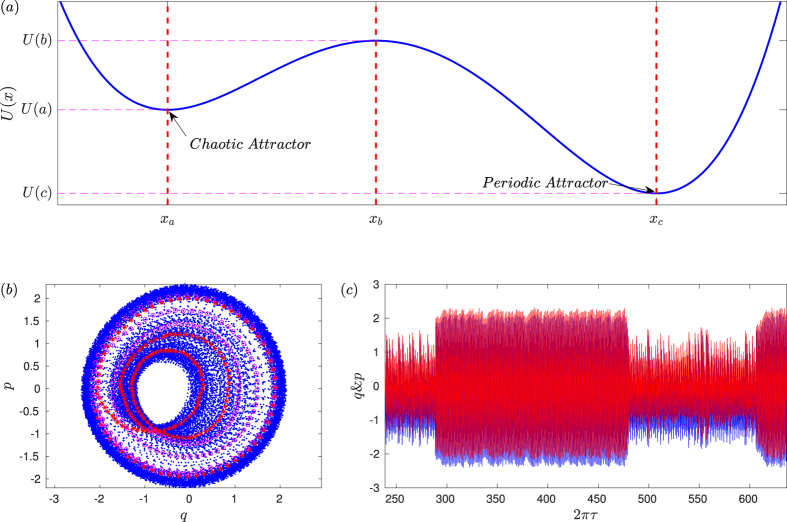
(**a**) A mechanical picture illustrating the noise-induced transition between chaotic and periodic attractors, where the periodic attractor is more stable than the chaotic attractor. For *g*_0_/*ω*_*m*_ = 0.056, representation in the *q* − *p* space (**b**), where the red stars represent the transition process from the inner chaotic attractor to the outer periodic attractor while the magenta circles represent the transition in the opposite direction. (**c**) The corresponding time series, where the blue and red colors are for *q* and *p*, respectively.

A natural question is whether the reverse process, i.e., transition from the periodic orbit to the chaotic orbit, can happen. In nonlinear dynamics, periodic attractors are usually more stable than chaotic attractors. Heuristically, a system in which a periodic and a chaotic attractors coexist can be viewed as particle motion in a mechanical system with two asymmetric potential wells subject to unbounded (e.g., Gaussian) noise, where the periodic attractor corresponds to the deep well and the chaotic attractor is associated with the shallow well, as schematically shown in Fig. 6(a). The probability for the particle to “hop” into the shallow well from the deep well is considerably smaller than that in the opposite direction. In optomechanical systems, this kind of backward transition can occur but it is rare. One such case is shown in Fig. 6(b,c), where the transition occurs at *g*_0_/*ω*_0_ = 0.056. For smaller values of *g*_0_, it is highly unlikely that the trajectory can switch into the periodic attractor. Even if this occurs, the probability for the trajectory to escape the periodic attractor will be exponentially small due to the higher potential barrier. For large values of *g*_0_, transition in both directions can occur, as shown in Fig. 6(c).

Our reasoning based on separating the deterministic and stochastic components of the Langevin equation does not depend on the specific details of the system, suggesting that the fast growing behavior in the average transient lifetime and the associated scaling law are *generic*.

## Discussion

To summarize, we investigate the fundamental problem of quantum-classical correspondence in optomechanical systems from the perspective of dynamical evolution. When the classical system exhibits chaos, the evolution of the quantum system contains two phases: chaotic motion in the (relatively) short time scale and regular motion in the long time scale. The transient chaotic behavior of the quantum system corresponds precisely to that in the classical limit - in this sense there is a well-defined quantum-classical correspondence. The long term behavior of the quantum system, however, is characteristically different from the classical behavior - in this sense there is a breakdown^25^ of the quantum-classical correspondence. As the classical regime is approached, the chaotic transient lifetime increases dramatically (faster than the Ehrenfest time for isolated systems - see below). Our finding of transient chaos in optomechanical systems, besides being a remarkable phenomenon by itself, provides a natural resolution for the paradoxical breakdown of quantum-classical correspondence.

In general, the problem of quantum-classical correspondence can be addressed through the approach of quantum-classical transition (QCT). It is known that, unlike special relativity where Einstein’s theory can be smoothly transformed to Newtonian mechanics in the limit *v*/*c* → 0, the approach of a quantum system to the classical limit *ħ* → 0 is singular. In the classical world, chaos exists in both dissipative and Hamiltonian systems, and chaotic dynamics are often studied in the phase space. However, to our knowledge, attempts to find chaos in the Schrödinger equation or in the quantum Liouville equation have not been convincingly successful. One reason is that isolated quantum systems are fundamentally linear. Another reason is that, the uncertainty principle forbids arbitrarily fine scale structures in the phase space. Indeed, in bounded and isolated (or closed) quantum systems the most complicated dynamics are quasiperiodic. Even though the transient behavior of a quantum system can be similar to that in the corresponding classical system, any classical features will be lost after a time scale called the Ehrenfest time: *t*_*E*_ ∝ *ħ*^−*δ*^, where *δ* is determined by the details of the system. Strictly, the Ehrenfest time holds for the idealized situation where the underlying system is fully closed. With the development of the quantum theory and advances in experimental techniques, the quantum dynamics of other types of situations have been considered, such as *unconditioned open* and *conditioned open* systems^66^,^67^. In the former case, the system is coupled to the environment but no information about the system is extracted, while for the latter information about the state of the system is extracted from it. For an unconditioned open system, the dynamical evolution is governed by the quantum Master equation, which is still linear. However, for a conditioned open system, its dynamical evolution follows a stochastic quantum equation that contains a nonlinear term representing the conditioning due to the measurement.

In the study of QCT, there are two general approaches to addressing the quantum-classical correspondence. The first is to focus on the agreement between the distribution functions, i.e., the quantum Wigner and the classical distribution functions - the weak form of QCT^26^,^27^. The second approach, the strong form of the QCT^67^,^68^, is to examine the localization of the quantum trajectory on the classical orbits, in which chaos can emerge naturally. To assess the degree of localization, continuous measurements of the system are required, introducing a nonlinear term in the quantum equation, so this approach is applicable only to conditioned open systems.

Table 1 presents an overview of the status of the knowledge about QCT, with knowns and unknowns specified. An outstanding issue concerns the scaling of the transition time in the strong QCT regime. In particular, the question is whether the QCT time follows the same scaling law as the Ehrenfest time. We address this issue in this paper by exploiting optomechanical systems subject to continuous heterodyne detection, which fundamentally exhibits a strong form of QCT. Qualitatively, our main finding is that transient chaos effectively serves as a bridge for the QCT. Quantitatively, we uncover a scaling law for the transition time which is different from that for the Ehrenfest time associated with the conventional QCT for isolated systems. With the advances in experimental techniques, there is now ability to observe quantum trajectories^25^,^29^. We expect the main results of this paper to be experimentally testable.

We make a few further remarks pertinent to our results.

### Remark 1: Transient chaos in quantum systems - what does it mean?

A quantum system is fundamentally linear. How can then a quantum trajectory be chaotic, even transiently? This paradox can be resolved, as follows. The Schrödinger (quantum Master) equation describes the time evolution of an individual system in an ensemble of identical systems, from which the mean value of any physical quantity, 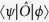 [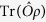] for an operator 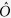, can be obtained. This is an ideal evolution process during which no further disturbance or measurement should be made; for otherwise the wavefunction will collapse into an eigenstate determined by the whole system, including the measurement apparatus. Based on the quantum trajectory theory, the time evolution of the mean value can also be produced from the QSD calculations through the ensemble average. When there is chaos in the classical limit, the ensemble averaging process can make the time evolution of the mean value periodic. That is, even when QSD calculation gives that a single quantum trajectory is chaotic in the transient phase, the ensemble average of many such trajectories can still be regular.

### Remark 2: Effect of measurement

A single quantum trajectory, however, is not physically meaningless. For a dissipative quantum system, the Master equation can yield the best prediction about the dynamical evolution of an ensemble of the system in absence of any measurement. The single trajectory calculated from quantum methods, such as QSD and quantum jump theory, has the physical meaning of conditioned realization of an individual system under a particular observation record, through homodyne/heterodyne detection and photodetector^69^,^70^. This makes the quantum trajectories *subjectively* real^70^. In the continuously conditioned measurement theory, any measurement introduces a factor called the detector efficiency, 0 ≤ *η* ≤ 1, into the QSD simulation^71^,^72^,^73^,^74^, which models the situation where the beam-splitter transmittivity is less than unity^71^. Mathematically, this factor can be taken into account by decomposing the photon fluctuation term into two uncorrelated terms of strength 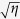 and 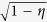, respectively^71^,^74^. In the limit *η* → 1, this form of stochastic equation is reduced to the equation simulated in our work, which corresponds to perfect detection.

### Remark 3: Effect of temperature

Our treatment of the breakdown of quantum-classical correspondence in optomechanical systems as a problem of strong QCT assumes the low temperature limit. As we argue and demonstrate, the transition from chaos to a regular state is mediated by quantum fluctuations or noise. Naturally we expect that thermal noise would play a similar role. In particular, if we focus on the classical system subject to thermal noise, a transition from a chaotic attractor to a periodic one can occur, accompanied by transient chaos.

In an optomechanical system, the fundamental physical constant *ħ* cannot be changed in experiments. The degree of quantum fluctuations can be controlled by adjusting or engineering other parameters, e.g., the mass of the cantilever, while keeping the system at low temperature. Weaker quantum fluctuations corresponding to smaller values of *g*_0_ can be realized using a heavier cantilever. In this case, the quantum system would behave chaotically for a relatively long time, due to the exponentially long transient lifetime. We note that, in the high temperature regime, the strength of the quantum fluctuations scales as 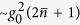, where 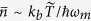. This indicates a counter-balancing effect between temperature 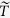 and mass *m* as ~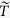/*m*. Consequently, at high temperatures a system of relatively large mass can still behave chaotically for a long time.

### Remark 4: Emergence of chaos in the quantum regime

In the works of Habib *et al*.^68^,^75^, the occurrence of chaos in the quantum regime was reported. The underlying mechanism lies in continuous measurement, which is key to resolving the quantum-classical correspondence, as demonstrated in our work.

A pertinent question is, since the transition time from classical chaotic to quantum regular motions can be quite short, why would there be chaos in the deep quantum regime as studied by Habib *et al*.? In these works, a toy model was studied and the main idea was to vary the effective Planck constant *ħ* (e.g., from 10^−2^ to 16) and the measurement strength *k* to calculate the quantum Lyapunov exponent *λ* and compare its values with those of the classical exponent *λ*_*Cl*_. It was found^68^,^75^ that, for *ħ* = 10^−2^, the classical results can be recovered through continuous changes in the measurement strength. Furthermore, non-negative values of *λ* were obtained before the classical limit, indicating that chaos may exist in the regime far away from the classical limit.

The optomechanical systems we studied are experimentally realizable, for which there are realistic criteria to determine if the system is in a classical or in a quantum regime. In particular, to characterize an optomechanical system, a number of key dimensionless parameters can be used - see, e.g., Eq. (119) in ref. 3. The most relevant parameter is *g*_0_/*κ* - the “quantumness” parameter, where *g*_0_/*κ* > 1 represents the strong couping regime in which the optical device can detect the change of even one phonon. In our work, we used *g*_0_/*κ* in the range 10^−2^~10^−1^. To determine whether this regime is quantum, we refer to Fig. 9 in ref. 3, which summarizes the experimental parameters for various quantum realizations. For example, in ref. 22, the parameter values *g*_0_/2*π* = 910 kHz and *κ*/2*π* = 500 MHz were used, which lead to *g*_0_/*κ* = 1.82 × 10^−3^. In ref. 76, the values *g*_0_/2*π* = 3.4 kHz and *κ*/2*π* ~ 1 MHz were used, giving rise to *g*_0_/*κ* ~ 10^−3^. In addition, in experimental studies of conditioned measurement of optomechanical systems^28^, the value of *g*_0_/*κ* used was about 10^−5^ but contributions from quantum noise were also taken into account. (In these experimental works, the aim was not finding chaos in the quantum regime.) Referring to the values of *g*_0_/*κ* realized in these experiments, we see that the systems studied in our work are in the quantum regime even for e.g., *g*_0_/*κ* = 0.045. Thus, there is no conflict between our result and that of Habib *et al*.^68^,^75^. Considering the fact that many quantum trajectories in our system localize on the classical chaotic attractor for thousands of periods and this time can be made significantly longer through small changes in the the parameter *g*_0_/*κ*, from the point of view of experiments, there is chaos in the quantum regime in our system. However, we emphasize that the main point of our work is not that we find chaos in the quantum regime. Our goal is to address the issue of quantum to classical transition quantitatively through a scaling analysis of the transition time. It is for this purpose that we use the notions of “classical limit” versus “quantum regime”.

### Remark 5: Appearance of chaos in the quantum regime in absence of classical chaos

There were recent reports of emergence of chaos in the quantum regime in absence of classical chaos^77^,^78^. In these works, the quantum versus classical “weights” of the system is controlled by the effective Planck constant, where the classical limit is reached when the constant approaches zero. In our system, the parameter *g*_0_ plays the same role. In classical nonlinear dynamical systems, chaos is common and noise can induce transition among different attractors - these phenomena are usually system and parameter dependent. A main point of our work is that quantum fluctuations can effectively serve as noise and induce transitions, with transient time depending on the fluctuation strength. We note that B. Pokharel *et al*.^77^ used quantum tunneling to explain their results. In our work we focused on the case where the classical limit is chaotic, and we observe transitions in both directions: from chaotic to regular motions and vice versa, and we argue that the transition probabilities in the opposite directions can be drastically different. In general, even for a set of parameters for which the asymptotic classical dynamics is regular, there can be transient chaos in relatively short time scales due to nonattracting chaotic invariant sets. When there is noise, there can be transitions between the regular attractor and the nonattracting chaotic set, leading to a combined chaotic attractor. In a general sense, quantum tunneling can induce transitions among different states and, as a result, chaos in the quantum regime can occur in open systems subject to continuous measurement. In this sense, our work does not contradict that of B. Pokharel *et al*.^77^.

### Remark 6: “Suppression” of classical chaos

Equation (2) holds in classical limit for which quantum fluctuations do not exist. In the quantum regime, the fluctuations are naturally incorporated into the quantum trajectory calculations. To account for the quantum fluctuations in the semiclassical theory, we use the quantum Langevin equation^52^. Note that Eq. (8) is a set of rescaled equations so that the fluctuation or “noise” strength is nothing but *g*_0_. However, the noisy version of Eq. (2) can be studied so as to reveal the equivalence between the effects of classical noise and quantum fluctuations. We focus on the noise-to-driving ratio, a quantity that increases with *g*_0_. In the quantum Duffing oscillator model^78^, when the parameter *β* is increased, the amplitude of the driving (*g*/*β*)cos(*ωt*) is reduced. For very large value of *β*, the quantum Lyapunov exponent becomes negative while the exponent in the classical limit remains positive, indicating a transition from chaos to a periodic behavior. We observed similar results in our optomechanical systems, i.e., the quantum fluctuations can suppress classical chaos.

Mathematically, suppression of classical chaos can be treated as a phenomenon of quantum fluctuation induced transition. In our optomechanical system the periodic attractor is apparently more stable than the chaotic attractor, which also appears to be the case in the quantum Duffing system studied by J. K. Eastman *et al*.^78^ in the parameter regime where there is chaos in the classical limit. For small values of *g*_0_ where the quantum fluctuations are weak, the classical chaotic behavior can last for a long time, as quantified by the scaling law uncovered in our paper. For relatively large values of *g*_0_, the transition time from chaos to a periodic behavior becomes significantly shorter. In our paper we also discuss the reverse transition and point out that the probability is negligibly small, as shown in Fig. 6(b,c). We attribute the suppression phenomenon to quantum fluctuations, as they are the main source of fluctuations in optomechanical systems.^10^,^12^,^20^.

## Methods

Historically, the method of quantum trajectory represented an efficient way to solve the master equation, and certain types of quantum trajectories can correspond to the result of conditioned measurement^29^,^30^,^31^,^32^,^33^,^34^,^35^,^36^,^37^,^38^,^39^,^40^. Mathematically, an ensemble of quantum systems whose state vectors are governed by a stochastic differential equation can have a density operator that satisfies a unique deterministic master equation. In contrast, a specific master equation can correspond to many different stochastic equations or different unravellings such as the QSD equation, the quantum jump equation, or the orthogonal jump equation^79^. While all the unravellings can be used to simulate the master equation, they have a different physical meaning. The most commonly calculated quantum trajectories are those from the QSD equation and the quantum jump equation, corresponding to homodyne and photon counting detection, respectively.

For a general Lindblad form of the master equation:


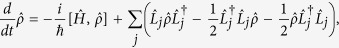


the QSD equation is^53^,^79^:


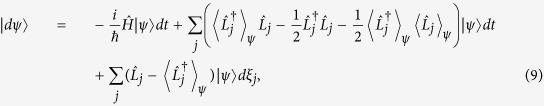


and the quantum jump equation is:


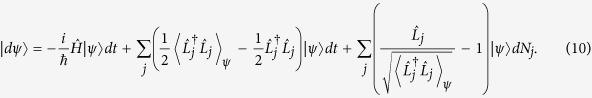


The QSD equation Eq. (9) is in the Ito form, which historically was called the nonlinear stochastic Langevin-Ito equation. Generally, for the Langevin equations of *N* variables of the form


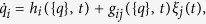


where {*q*} = *q*_1_, *q*_2_, …, *q*_*N*_ and 〈*ξ*_*i*_(*t*)〉 = 0, 〈*ξ*_*i*_(*t*)*ξ*_*j*_(*t*′)〉 = 2*δ*_*ij*_*δ*(*t* − *t*′), the corresponding probability density function *W*({*x*}, *t*) satisfies the Fokker-Planck equation^80^


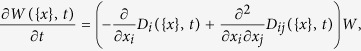


where the drift and diffusion coefficients are defined as


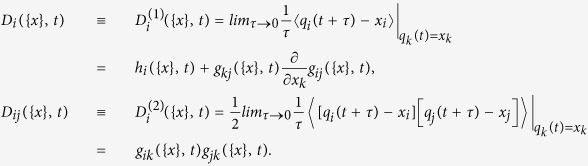


Note that *q*_*i*_(*t* + *τ*)(*τ* > 0) is a solution of the Langevin equation, which has the sharp value *q*_*k*_(*t*) = *x*_*k*_ (*k* = 1, 2 …, *N*) at time *t*. The quantity 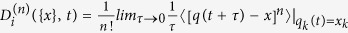 is the Kramers-Moyal expansion coefficients. For a process described by the Langevin equation with *δ*-correlated Gaussian noise, all the Kramers-Moyal coefficients *D*^(*n*)^ with *n* ≥ 3 vanish^80^. The physical significance is that the deterministic component of the Langevin equations contributes to the drift part in the evolution of the probability distribution while the stochastic component contributes to both the drift and diffusion evolution of the probability distribution.

In general, QSD represents a conditioned measurement experiment and the wave functions that it generates are normally localized about a point in the phase space. This fact can be exploited to improve the computational efficiency^81^. Say a wave function is localized about the point (*q*, *p*). We can represent it using the so-called *excited coherent* basis states, |*q*, *p*, *n*〉 = *D*(*q*, *p*)|*n*〉, instead of a large number of Fock states. Physically, this means that we exploit a moving basis that separates the wavefunction representation into a classical part (*q*, *p*) and a quantum part |*q*, *p*, *n*〉, which is effectively a *mixed* representation. The excited coherent states are defined through the coherent state displacement operator:


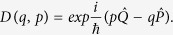


where 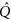 and 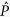 are the position and momentum operators. The displacement operator can be defined using the creation/annihilation operator as


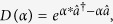


and the matrix element in Fock state is


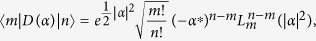


where 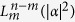 is the associate Laguerre polynomials.

Suppose at *t* = *t*_0_ the state of the system is localized about (*q*_0_, *p*_0_), i.e.,


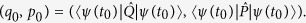


After one time step, we have


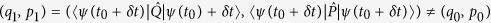


We then shift the basis from (*q*_0_, *p*_0_) to (*q*_1_, *p*_1_), which can be done through


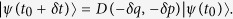


Besides the wavefunction, we need to transform the operators into the new basis as well. The procedure is straightforward due to certain properties of the displacement operator:


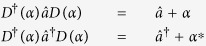


which changes the transformation of the Hamiltonian and the operators from two matrix multiplications to one matrix addition. In spite of the need to perform base transformation at each time step, the overall computational speed is faster than that with the Fock state calculation.

## Additional Information

**How to cite this article**: Wang, G. *et al*. Transient chaos - a resolution of breakdown of quantum-classical correspondence in optomechanics. *Sci. Rep.* **6**, 35381; doi: 10.1038/srep35381 (2016).

## Figures and Tables

**Figure 1:**
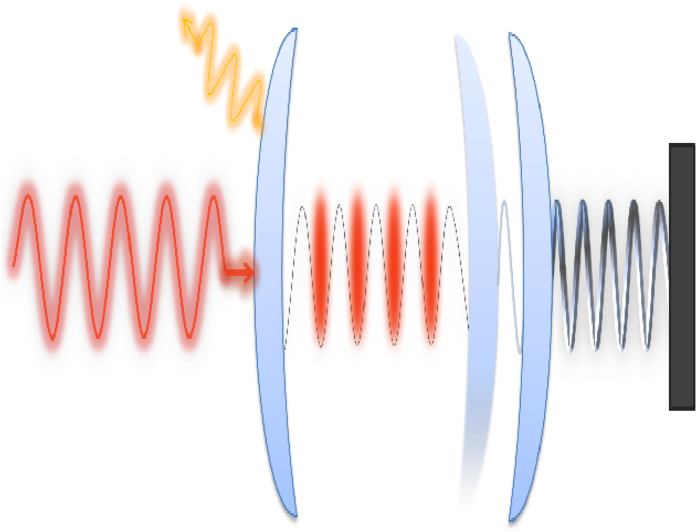
A schematic figure of the optomechanical system.

**Table 1:** An overview of distinct QCT regimes.

	Conventional QCT	Weak QCT	Strong QCT
System	Isolated	Unconditioned Open	Conditioned Open
Equation	Schrödinger	Master	Quantum Trajectory
Dynamics	Linear	Linear	Stochastic Nonlinear
Characteristic Time	Ehrenfest time ~*ħ*^−*δ*^	Unknown at present	Eq. (7) *discovered in this paper*
